# Supplementary material for: Dynamic Training Enhances Machine Learning Potentials for Long-Lasting Molecular Dynamics
Source: J Chem Inf Model. 2025 Jul 22;65(15):8033–41. doi: 10.1021/acs.jcim.5c01180 (PMC12344694; doi:10.1021/acs.jcim.5c01180)
Supplement: Supplementary file 1 [file ci5c01180_si_001.pdf]

Supporting Information: Dynamic Training Enhances Machine Learning Potentials for Long-Lasting Molecular Dynamics

Ivan Žugec,[*,†,‡] Tin Hadži Veljković,[¶] Maite Alducin,[*,†,§] and J. Iñaki Juaristi[*,‡,†,§]

†*Centro de Física de Materiales CFM/MPC, CSIC-UPV/EHU, Paseo Manuel de Lardizabal 5, 20018, Donostia-San Sebastián, Spain*

‡*Departamento de Polímetros y Materiales Avanzados: Física, Química y Tecnología, Facultad de Química (UPV/EHU), Apartado 1072, Donostia-San Sebastián, 20080, Spain*

¶*UvA-Bosch Delta Lab, University of Amsterdam, Amsterdam Science Park 904, Amsterdam, 1098 XH, Netherlands*

§*Donostia International Physics Center, Paseo Manuel de Lardizabal 4, Donostia-San Sebastián, 20018, Spain*

E-mail: zugec.ivan@gmail.com; maite.alducin@ehu.eus; josebainaki.juaristi@ehu.eus

Supplementary Note 1

In order to benefit from performing dynamics during the training process, every operation from the first model prediction up to weight adjustments has to be differentiable. This presents a problem when building the atomic neighborhoods within the S-loop shown in Fig. 1**b** in the main text that stems from the binary nature of operations typically used to construct it. One way to deal with this problem is to extract neighborhood information of

each atomic structure from the underlying AIMD simulations and record it during the data preprocessing step. Given sufficiently accurate predictions of atomic forces, the neighborhood structure of updated atomic positions will match the one from corresponding AIMD simulations. This is why it is very important to converge the model on subsequence length equal to one before introducing larger lengths in the training process.

Storing neighborhood structures at each step did not exceed our computational limits. For this reason, this is the strategy that we followed in this work. The neighborhood structure was updated at every step according to the corresponding neighborhood structure in the AIMD simulations.

However, applying the DT method to large datasets and systems with large amount of atoms could increase the computational cost. In other words, for too large systems and too large datasets, storing the AIMD neighboorhood structures in order to update the neighboorhood structure at every step of the S-loop could be prohibitive. This is why we propose the following idea to alleviate the computational burden. If we assume that the changes in the neighborhood structure occur gradually along the dynamics, we can reuse the same neighborhood structure for multiple simulation steps. To examine the validity of this approximation for the system used in this work, we measure the rate of change of the neighborhood structure for atomic configurations offset by a different amount of simulation steps Δt. As a measure of a difference between sets of neighbors we use the Jaccard similarity index

$$J\left(N(i,t),\, N(i,t+\Delta t)\right) = \frac{|N(i,t) \cap N(i,t+\Delta t)|}{|N(i,t) \cup N(i,t+\Delta t)|}, \tag{S1}$$

where $N(i,t)$ is the set of neighbors of atom i at simulation time t. The closer Δt is to zero, the closer the Jaccard index is to unity. The averaged Jaccard index for each atom over all 100 AIMD simulations of the training set is shown in Fig. S1. Indices 0 and 1 correspond to hydrogen atoms forming the H_2 molecule, indices from 2 to 50 correspond to carbon atoms, and indices 51 to 56 correspond to palladium atoms. As we would expect,

hydrogen atoms have the lowest similarity index as they move the most. However, note that after nine simulation steps the averaged similarity is still above 93% for hydrogen atoms and above 97% for the rest of atoms in the system. Moreover, training models with neighborhood updates taking place every five steps yielded comparable performance. This framework can therefore serve as a systematic method to determine appropriate neighborhood-structure-update frequencies across different systems in the dataset.

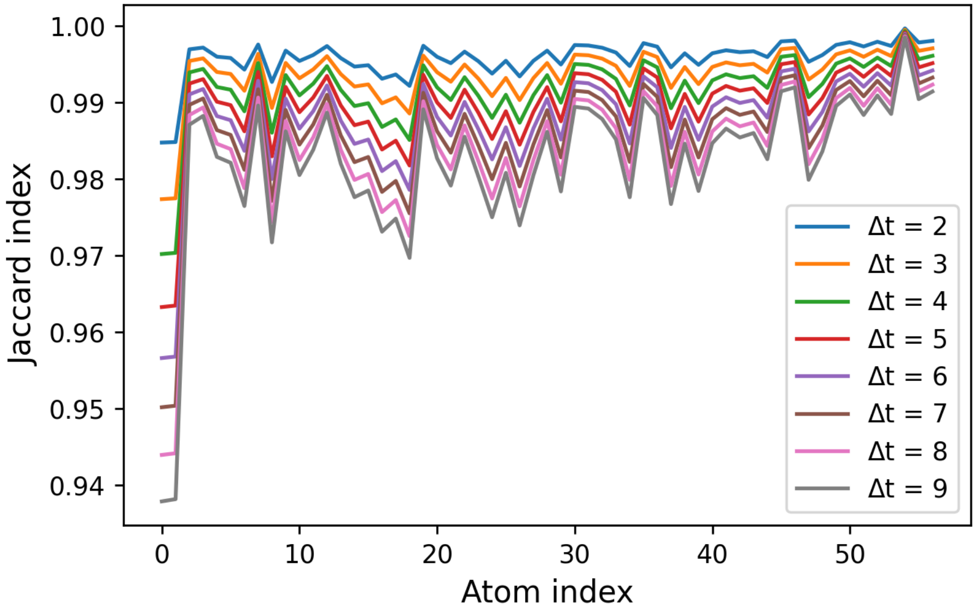

Figure S1: Jaccard index of neighborhood structure as a function of atom index in the $H_2Pd_6@G_{vac}$ for various offsets Δt. Indices 0 and 1 correspond to hydrogen atoms forming the H_2 molecule, indices from 2 to 50 correspond to carbon atoms, and indices 51 to 56 correspond to paladium atoms.

The results of Fig. S1 show that in the case of our system, instead of updating the atomic neighborhoods at every step, we could have opted for updating them after several steps. They also suggest that this could be the ideal strategy in case of too large systems for which updating at every step would be prohibitive. Note that, the main reason for this is that in well converged AIMD simulations the neigborhood structure varies smoothly. For example, the hydrogen atoms in the aforementioned AIMD simulations explore very different situations and neighborhoods (forming an isolated H_2 molecule far from the Pd cluster, forming a H_2

molecule interacting strongly with the Pd atoms, dissociated H atoms interacting with the Pd atoms). Still, the variation of the neighborhood structure for these H atoms along the dynamics is smooth enough to allow for updating neighborhood structure after several steps. This constitutes a strong indication of the validity of the proposed strategy for more general and larger systems of atoms.

Supplementary Note 2

Here we show how the choice of the loss function directly impacts the NNP performance during extended molecular dynamics simulations. The comparison involves three models: EGNN-MEPA, EGNN-MAE, and EGNN-MSE named after the loss functions used during training process. Weighted MSE loss function, used to train MACE as well as EGNN-MSE, and MEPA are already defined in the Methods section of the main text. Mean absolute error (MAE) loss function is defined as

$$L_{\mathrm{MAE}} = \frac{1}{B}\sum_{b}^{B}\frac{1}{N_b}\left|E_{\mathrm{pred},b} - E_{\mathrm{DFT},b}\right| + \frac{1}{B}\sum_{b}^{B}\frac{1}{3N_b}\sum_{i=1}^{N_b}\sum_{\alpha=1}^{3}\left|F_{\mathrm{pred},\alpha,b} - F_{\mathrm{DFT},\alpha,b}\right|. \tag{S2}$$

To ensure a controlled comparison, all NNPs have identical architectures and equal number of trainable parameters. Moreover, all of them were trained on the same training and validation sets. Figs. S2–S4 show the components of MEPA during the training process on structures present in the validation set for each atomic species (carbon, palladium, and hydrogen) present in the system. Even though the validation error given to the scheduler during the training process was consistent with the loss function for each respective model, we calculate the MEPA for each model in order to compare them. Models trained with MAE and MSE loss functions show better performance on carbon and palladium atoms, but perform significantly worse for hydrogen atoms. This is because MAE and MSE focus on minimizing the errors of the whole structure, while MEPA focuses on minimizing the error of each atom species.

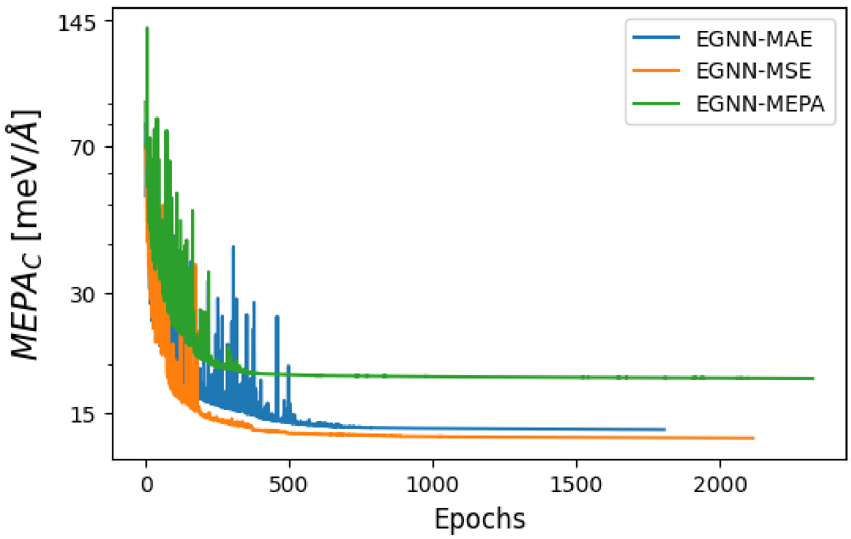

Figure S2: Contribution to MEPA coming from carbon atoms as a function of epochs, calculated from atomic structures present in the validation set. All models, namely, EGNN-MAE (blue), EGNN-MSE (orange), and EGNN-MEPA (green), were trained on 217,478 training structures and validated on 11,447 structures.

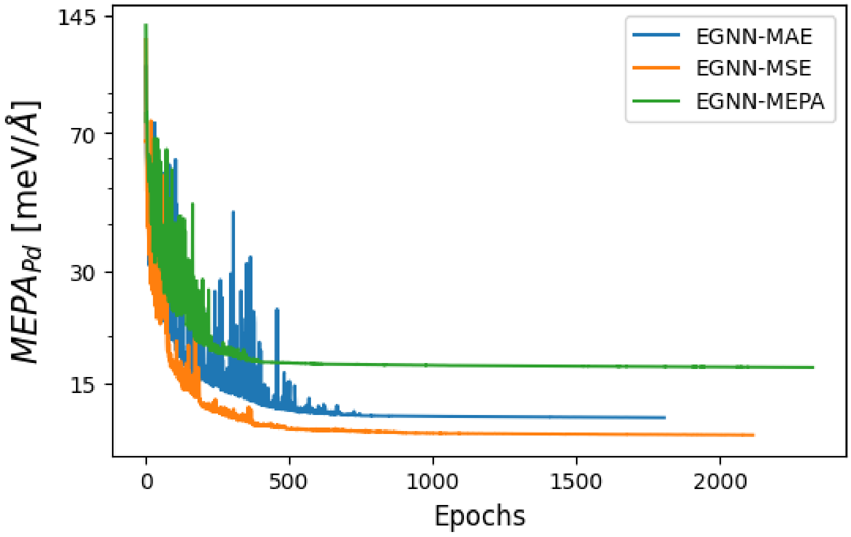

Figure S3: Contribution to MEPA coming from palladium atoms as a function of epochs, calculated from atomic structures present in the validation set. All models, namely, EGNN-MAE (blue), EGNN-MSE (orange), and EGNN-MEPA (green), were trained on 217,478 training structures and validated on 11,447 structures.

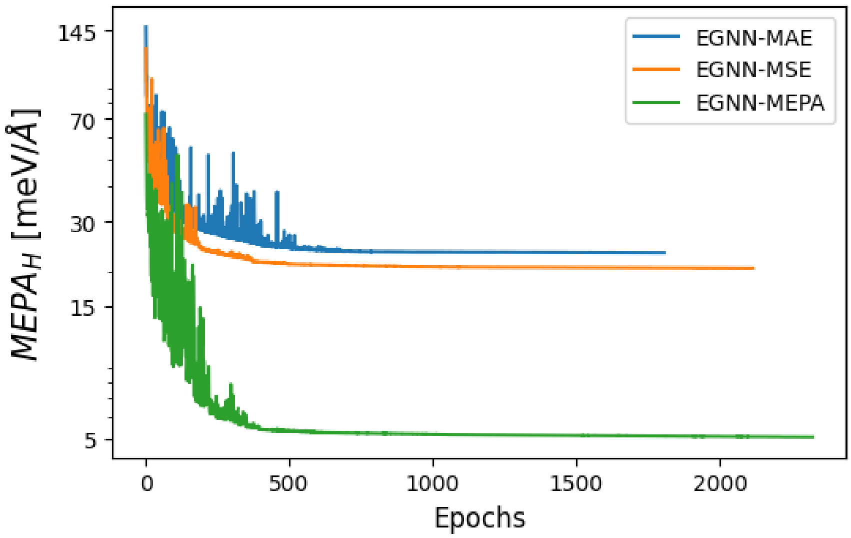

Figure S4: Contribution to MEPA coming from hydrogen atoms as a function of epochs, calculated from atomic structures present in the validation set. All models, namely, EGNN-MAE (blue), EGNN-MSE (orange), and EGNN-MEPA (green), were trained on 217,478 training structures and validated on 11,447 structures.

Supplementary Note 3

Here we demonstrate the ability of DT-EGNN to capture a complex dynamical feature of the H_2Pd_6@Gvac system that typically emerges only after thousands of integration steps. In Ref. S1 it was shown that hydrogen-dissociation events are often accompanied by a structural rearrangement of the palladium cluster. More precisely, the dissociation of H_2 in the Pd cluster can induce its transition from the original octahedral structure to an incomplete pentagonal bypiramid structure. It was shown that the structural transition basically consists in the drastic elongation of the Pd-Pd distance between two particular Pd neighbors in the equatorial plane of the octahedron. It is worth mentioning that this structural change is a relatively long time process that takes place 1-3 ps after H_2 dissociation.

In order to test whether we can observe such events, we used the same initial conditions as the corresponding AIMD calculations and, using DT-EGNN as PES, propagated those initial conditions for 4 ps with the integration time step equal to 0.1 fs. Note that this requires performing 40 000 integration steps, to be compared with the 11 steps utilized in the longest training subsequences. The obtained results suggest that not only do we observe events featuring dissociation of the hydrogen molecule, but also reproduce the structural transition of the palladium cluster. Fig S5 shows the time evolution of the distance between the two relevant palladium atoms for both the DT-EGNN NNP and the AIMD reference. Although a sample of 100 trajectories is insufficient for statistically meaningfull discussion of dissociation probabilities (10 events for DT-EGNN vs. 7 for AIMD), it is noteworthy that three of these events (trajectories 6, 20 and 97) share identical initial conditions. All in all, these results show the good performance of the method on long time dynamics and its ability to describe complex dynamical phenomena.

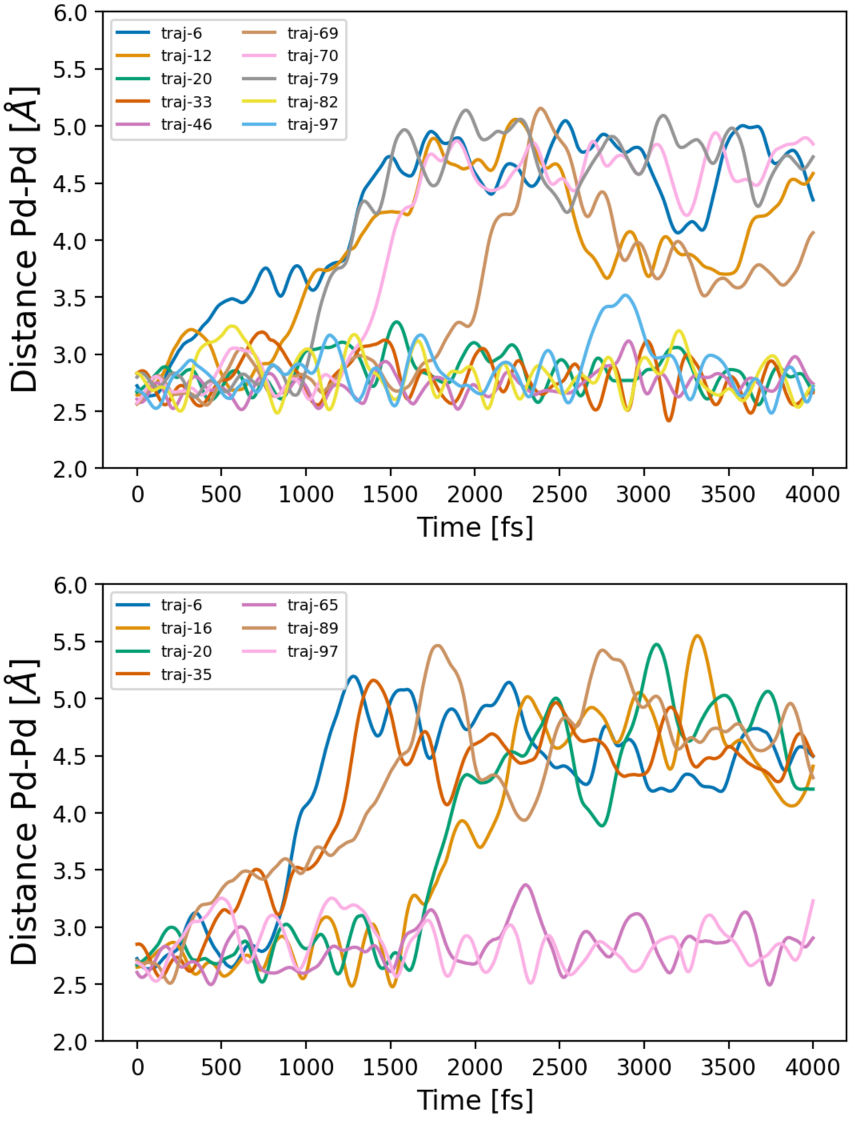

Figure S5: Distance between the two Pd atoms exhibiting pronounced elongation along the trajectories corresponding to dissociative adsorption of H_2 for DT-EGNN NNP (top) and AIMD (bottom).

Supplementary Note 4

Here we summarize the information on all DFT and computational settings that were employed in Ref. S1 to run the AIMD simulations, from which we obtained the dataset used in the present manuscript.

The calculations were based on spin-polarized DFT for a H_2 molecule impinging on a Pd_6 cluster anchored to a graphene vacancy (the system denoted as $Pd_6@G_{vac}$ in the main text). The plane waves based VASP code[S2,S3] was used to perform the calculations and exchange correlation effects were described using the generalized gradient approximation PW91 functional.[S4] The interaction of the explicitly considered valence electrons with the atomic cores was treated in the projector augmented wave (PAW) approximation,[S5] for which the PAW potentials supplied with the VASP package were used.[S6] The energy cutoff for the plane-wave basis set was 400 eV. The integration in the Brillouin zone was performed using a Γ-centered 2×2×1 Monkhorst-Pack grid of special **k**-points.[S7] The first-order Methfessel-Paxton broadening scheme, with a 0.1 eV width, was used to consider fractional electronic-state occupancies.[S8]

The supercell consisted of a hexagonal 5×5 graphene layer in the lateral directions. The height of the supercell in the normal direction to the graphene layer was 14 Å. The substrate was relaxed until the forces on each Pd and C were below 0.02 eV/Å.

The ab initio molecular dynamic simulations used to generate the neural network potential energy surface in the present work were obtained for a H_2 molecule impinging on $Pd_6@G_{vac}$ under normal incidence conditions, with an initial substrate temperature of 300 K. The zero pressure limit was studied by considering one H_2 per simulated trajectory. The initial translational and vibrational energies of the H_2 molecule were $E_i = 0.125$ eV and $E_{vib}(\nu = 0, j{=}0) = 0.27$ eV, respectively. The center of mass of the molecules was initially located at a height 9 Åabove the graphene layer. The initial lateral position of the center of mass and the orientation of the molecule were randomly sampled. Before running the AIMD simulations, the $Pd_6@G_{vac}$ substrate was equilibrated at 300 K for 7 ps using the Nosé

thermostat.[S9] Sunsequently, the energy-conserving AIMD simulations of the H_2 molecules impinging on the anchored cluster were performed, taking the initial positions and velocities of the C and Pd atoms randomly from the set of configurations generated during the thermalization at $T = 300$ K. The number of calculated trajectories was 100, the integration time step 0.5 fs, and the total integration time was 4 ps.

Supplementary Note 5

Here, we show that our DT-EGNN NNP can perform molecular dynamics simulations lasting several orders of magnitude longer than those encountered during training, while maintaining the stability of the system. Additionally, we provide direct evidence for improved stability as the subsequence length, used during training process, increases.

Using DT-EGNN ($S = 11$) as a NNP, 100 MD simulations with the integration time step of 0.5 fs and total time of 100 ps were performed. Taking the last snapshot of each of the 100 trajectories, we calculate distances between all atoms in the system and present them as a histogram. Furthermore, for comparison, we do the exact same thing for 100 simulations performed with AIMD. A comparison of normalized densities of interatomic distances between these two methods is shown on Fig. S6.

It is important to stress that the histogram for DT-EGNN is created for the atomic configurations at the end of 100 ps, while AIMD simulations last between 800 fs and 4 ps. Still, almost all of the peaks present in the AIMD structures are also present after 100 ps, providing compelling evidence that our DT-EGNN approach reliably maintains structural stability well beyond the subsequence lengths used during training.

Finally, we also performed 100 MD simulations using DT-EGNN at subsequence length equal to one ($S = 1$) and compared the resulting distribution of interatomic distances at 100 ps to those obtained from AIMD simulations (see Fig. S7). Relative to DT-EGNN at $S = 11$, results for DT-EGNN at $S = 1$ suggest that the discrete peaks of interatomic distances are

less obvious for this distribution. Furthermore, significant portion of interatomic distances lay outside of the range of the AIMD distribution (very small as well as very big interatomic distances) suggesting much stronger deviations from the initial structure.

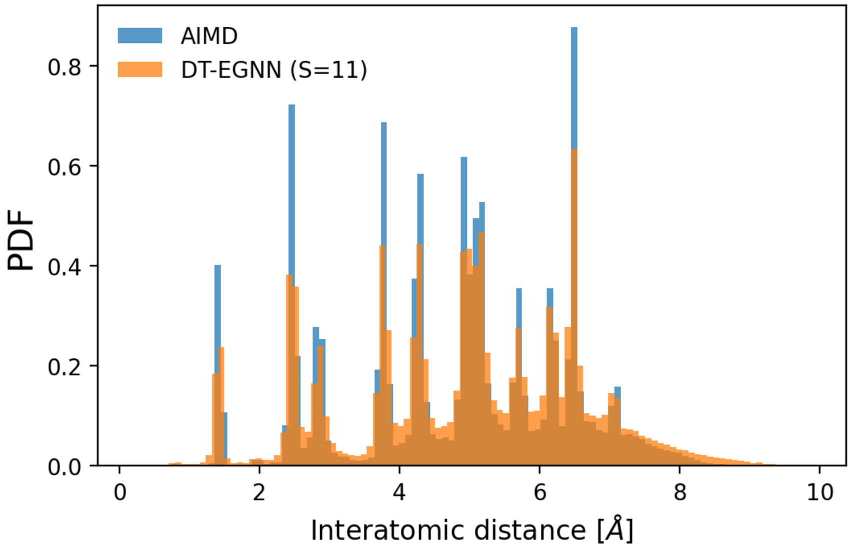

Figure S6: Comparison of normalized histograms of interatomic distances extracted from 100 independent MD trajectories generated by DT-EGNN ($S = 11$, orange, snapshots at 100 ps) and 100 AIMD trajectories (blue, durations 800 fs–4 ps).

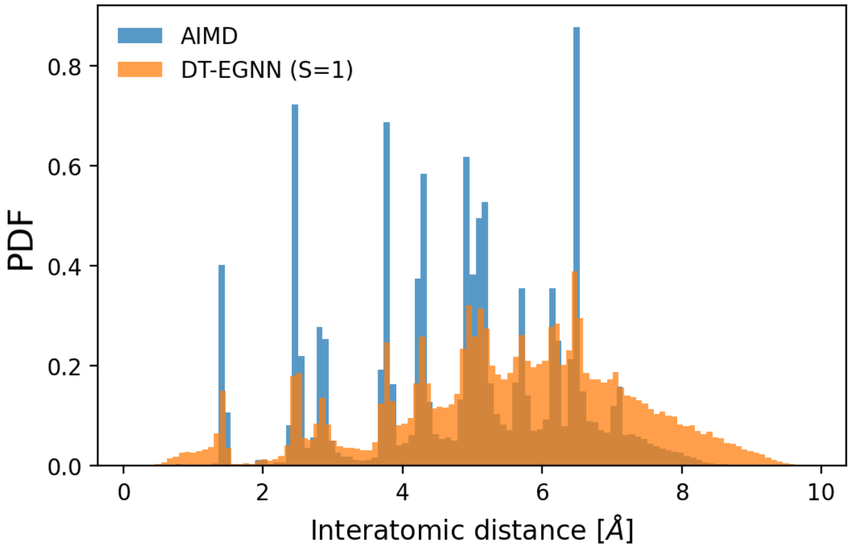

Figure S7: Comparison of normalized histograms of interatomic distances extracted from 100 independent MD trajectories generated by DT-EGNN ($S = 1$, orange, snapshots at 100 ps) and 100 AIMD trajectories (blue, durations 800 fs–4 ps).

Acknowledgements

Financial support provided by the Spanish MCIN/AEI/10.13039/501100011033/, FEDER Una manera de hacer Europa (Grant No. PID2022-140163NB-I00), Gobierno Vasco-UPV/EHU (Project No. IT1569-22), and the Basque Government Education Departments' IKUR program, also co-funded by the European NextGenerationEU action through the Spanish Plan de Recuperación, Transformación y Resiliencia (PRTR). Computer resources were provided by the Donostia International Physics Center (DIPC) Supercomputing Center.

References

(S1) Alducin, M.; Juaristi, J. I.; Granja-DelRío, A.; López, M. J.; Alonso, J. A. Dynamics of cluster isomerization induced by hydrogen adsorption. *J. Phys. Chem. C* **2019**, *123*, 15236–15243.

(S2) Kresse, G.; Furthmüller, J. Efficiency of ab-initio total energy calculations for metals and semiconductors using a plane-wave basis set. *Computational Materials Science* **1996**, *6*, 15–50.

(S3) Kresse, G.; Hafner, J. Ab initio molecular-dynamics simulation of the liquid-metal–amorphous-semiconductor transition in germanium. *Phys. Rev. B* **1994**, *49*, 14251–14269.

(S4) Perdew, J. P.; Chevary, J. A.; Vosko, S. H.; Jackson, K. A.; Pederson, M. R.; Singh, D. J.; Fiolhais, C. Atoms, molecules, solids, and surfaces: Applications of the generalized gradient approximation for exchange and correlation. *Phys. Rev. B* **1992**, *46*, 6671–6687.

(S5) Blöchl, P. E. Projector augmented-wave method. *Phys. Rev. B* **1994**, *50*, 17953–17979.

(S6) Kresse, G.; Joubert, D. From ultrasoft pseudopotentials to the projector augmented-wave method. *Phys. Rev. B* **1999**, *59*, 1758–1775.

(S7) Monkhorst, H. J.; Pack, J. D. Special points for Brillouin-zone integrations. *Phys. Rev. B* **1976**, *13*, 5188–5192.

(S8) Methfessel, M.; Paxton, A. T. High-precision sampling for Brillouin-zone integration in metals. *Phys. Rev. B* **1989**, *40*, 3616–3621.

(S9) Nosé, S. A unified formulation of the constant temperature molecular dynamics methods. *The Journal of Chemical Physics* **1984**, *81*, 511–519.
